# Advancements in Pan-Canadian Data Access and Analysis Facilitation: Insights from Collaborative Health Research supported in Alberta, British Columbia, and Ontario

**DOI:** 10.23889/ijpds.v9i5.2848

**Published:** 2024-09-18

**Authors:** Stefana Jovanovska, Joanna Ou, Erik Youngson, Tim Choi, Carmen La, Erind Dvorani, Refik Saskin, Michael Paterson, Victoria Kirsh, Philip Awadalla, Jennifer Brooks, Sheraz Cheema, Nouar Elkhair, Jennifer Vena, Shandra Harman, Kelly McDonald, Parveen Bhatti, Dina Skvirsky

**Affiliations:** 1 ICES; 2 Health Data Research Network; 3 Alberta Health Services; 4 Population Data BC; 5 Ontario Institute for Cancer Research; 6 CanPath; 7 Alberta Health Services - Alberta’s Tomorrow Project; 8 Ontario Institute for Cancer Research - Ontario Health Study; 9 Cancer Imaging BC Cancer Research Centre -BC Generations Project

## Objective

To highlight progress in facilitating and supporting pan-Canadian data analysis research that is supported by a central coordinating center. This process will be illustrated with the use of a recently completed project that has been supported using data from Alberta, British Columbia, and Ontario..

## Approach

The project represents a significant endeavor within the central coordinating center, as it necessitates coordination for data access, data importation and analytical support across three provinces. The focus will be on the administrative processes refined to support collaborative research endeavors. While specific project details will remain undisclosed, procedural enhancements within the central coordinating center framework will be highlighted. Key discussion points include standardized protocols for data access, collaborative efforts to streamline data importation, and facilitation of analytical support across jurisdictions. The type of cross-jurisdictional analytic support will as well be highlighted; data variables harmonization and sharing of data algorithms cohorts across the participating provincial data centers in support of the meta-analysis.

## Results

The successful completion of the final data analysis underscores the effectiveness of a unified data access coordination center for researchers seeking multi-regional health data in Canada. By highlighting advancements enabled by the central coordinating center and provincial data centers, this submission aims to inform researchers about the current landscape of pan-Canadian research and foster opportunities for future collaboration.

## Implications

Sharing insights and lessons from this project emphasizes the advancements facilitated by the central coordinating center and provincial data centers, informing researchers about the potential of pan-Canadian research, and encouraging future collaborative endeavors.

